# Frequencies of Combined Dysfunction of Cytochromes P450 2C9, 2C19, and 2D6 in an Italian Cohort: Suggestions for a More Appropriate Medication Prescribing Process

**DOI:** 10.3390/ijms241612696

**Published:** 2023-08-11

**Authors:** Giovanna Gentile, Ottavia De Luca, Antonio Del Casale, Gerardo Salerno, Maurizio Simmaco, Marina Borro

**Affiliations:** 1 Department of Neurosciences, Mental Health and Sensory Organs (NESMOS), Sapienza University, Via di Grottarossa 1035/1039, 00189 Rome, Italy; giovanna.gentile@uniroma1.it (G.G.); gerardo.salerno@uniroma1.it (G.S.); maurizio.simmaco@uniroma1.it (M.S.); 2 Laboratory of Clinical Biochemistry, Advanced Molecular Diagnostic Unit, Sant’Andrea University Hospital, Via di Grottarossa 1035/1039, 00189 Rome, Italy; 3 Department of Dynamic and Clinical Psychology and Health Studies, Faculty of Medicine and Psychology, Sapienza University of Rome, 00189 Roma, Italy; antonio.delcasale@uniroma1.it; 4 Unit of Psychiatry, Sant’Andrea University Hospital, Via di Grottarossa 1035/1039, 00189 Rome, Italy

**Keywords:** cytochrome P450, CYP2C9, CYP2C19, CYP2D6, drug–drug interactions, pharmacogenomics, polypharmacy, co-prescription, adverse drug reactions

## Abstract

Improper drug prescription is a main cause of both drug-related harms (inefficacy and toxicity) and ineffective spending and waste of the healthcare system’s resources. Nowadays, strategies to support an improved, informed prescription process may benefit from the adequate use of pharmacogenomic testing. Using next-generation sequencing, we analyzed the genomic profile for three major cytochromes P450 (CYP2C9, CYP2C19, CYP2D6) and studied the frequencies of dysfunctional isozymes (e.g., poor, intermediate, or rapid/ultra-rapid metabolizers) in a cohort of 298 Italian subjects. We found just 14.8% of subjects with a fully normal set of cytochromes, whereas 26.5% of subjects had combined cytochrome dysfunction (more than one isozyme involved). As improper drug prescription is more frequent, and more burdening, in polytreated patients, since drug–drug interactions also cause patient harm, we discuss the potential benefits of a more comprehensive PGX testing approach to support informed drug selection in such patients.

## 1. Introduction

Cytochrome P450 (CYP450) isozymes constitute a large superfamily of heme proteins involved in various metabolic pathways such as bile acid biosynthesis, cholesterol and steroid hormone metabolism, and vitamin metabolism [1]. CYP450s became popular after their key role in the pre-emptive prediction of individual response to drug administration was revealed.

Drug metabolism is determined by the number and type of biochemical transformations the drug goes through inside the human body, determining the pharmacokinetics. Drug biotransformations are performed by a plethora of enzymes, grouped into Phase I (oxidative, reductive, and hydrolysis reactions) and Phase II (conjugation reactions) drug metabolic enzymes (DMEs). DME action produces drug metabolites that can be more easily eliminated and may have or not have pharmacological/toxic action. Thus, the amount of circulating drug/metabolites, which mediates both pharmacological actions (e.g., efficacy) and undesirable effects (side effects, adverse drug reactions), is mainly dependent on the activity level of DMEs, which often show extensive interindividual variability. Such variability has two main sources: (i) genetic variability, affecting the quality or quantity of the expressed protein, and (ii) biochemical enzyme regulation by inducing or inhibiting molecules. Both mechanisms contribute to an increased range of interindividual variability in the activity level of the Phase I enzymes CYP450s, which metabolize up to 80% of available drugs by families 1, 2, and 3 [2,3].

Genetic variability leading to heavily decreased/increased activity of CYP450s (and other pharmacogenes) and its effect on drug efficacy/toxicity are studied by pharmacogenomics (PGXs). PGX studies have led to an extensive comprehension of the genotype effects on individual drug response, and this knowledge has been widely systemized by international scientific consortia, leading to the development of guidelines and recommendations about the opportunity to characterize CYP450 gene variants and about the actions to take (such as drug dose adjustment or drug substitution) when a patient carries these variants [4,5]. Pre-emptive PGX testing, i.e., a DNA test aimed to evaluate the presence of clinically relevant variations (polymorphisms) in genes involved in drug response, with the aim to prevent adverse drug reactions (ADRs) and/or inefficacy, is compulsory (e.g., recommended in the drug label) for an increasing but still limited list of medications [6]. Official recommendations are released only when wide and comprehensive data on genotype–phenotype associations are available—thus, only when large and well-designed studies have been completed. This is usually the case for medications associated with high therapeutic risk, such as anticancer drugs or anticoagulant medications [7,8]. The main cytochromes included in PGX testing recommendations by the U.S. Food and Drug Administration (FDA) are CYP2C19 (25 drugs in 10 therapeutic areas), CYP2C9 (17 drugs in 9 therapeutic areas), and CYP2D6 (72 drugs in 13 therapeutic areas) (Table 1). Since genes encoding CYP450s are often highly polymorphic, such recommendations refer to the “genotype-predicted” phenotype, such as poor metabolism (PM), intermediate metabolism (IM), normal metabolism (NM), or rapid/ultra-rapid metabolism (UM).

The biochemical modulation of CYP450 proteins is an extremely variegate phenomenon since a long list of inducers or inhibitors exists, including many drugs themselves, food and beverages, and smoking [9,10]. The network of drug–CYP450 interactions, besides determining the pharmacokinetics of a substrate drug, contributes to the phenomenon of drug–drug interactions (DDIs): modulating the activity level of a CYP450 isozyme, a drug can alter the pharmacokinetics of a second drug metabolized by the same CYP450. The more drugs are taken together, the higher the risk of deleterious DDIs, which, in turn, affects the treatment’s efficacy, safety, and compliance [11,12]. Nowadays, DDI evaluation is increasingly affordable and advisable, since many bioinformatics tools (free or commercial) have been established to check DDIs and to optimize pharmacological therapies by drug exchange/dose adjustment [13,14,15]. However, it is noteworthy that DDI analysis tools usually suppose a normal activity of DMEs, whereas the DDI profile of a drug cocktail should be corrected by the genomic profile of each patient. For example, in a CYP2D6 normal metabolizer, DDI analysis could recommend avoiding the co-administration of a substrate and an inhibitor of the enzyme, but such a warning would be much stronger for a CYP2D6 poor metabolizer and weaker for a CYP2D6 rapid metabolizer. The landscape becomes more and more complex according to the number of drugs and to the number of dysfunctional (PM, IM, or UM) DMEs in a given subject.

In our opinion, the time is ripe to drive a paradigm shift in the current approach to DDI analysis and PGX testing, enlarging the target patient population where PGX testing is recommendable. We especially refer to the number of polytreated patients, which includes main categories such as elderly patients (high frequency of comorbidities to be treated), oncologic patients (high frequency of co-medications to treat cancer therapy’s side effects), and psychiatric patients (high frequency of co-prescription to reach therapeutic response). In these groups, comprehensive PGX testing (e.g., including main pharmacogenes) should be preemptively performed to guide appropriate and patient-sized selection of the main drug(s) (e.g., medication to treat the main disease) and to harmonize co-prescriptions according to the specific DDI/PGX pattern. The proposed approach is increasingly actionable considering the advent, diffusion, and increasing economical sustainability of high-throughput technologies (determining many genetic variants in a single test) applied to PGX testing, such as next-generation sequencing [16,17].

This study was aimed to estimate the extent of potential benefits of pre-emptive and comprehensive PGX testing, by analyzing the combined frequencies of dysfunctional alleles for three main cytochromes P450 (2C9, 2C19, and 2D6) in a cohort of 298 Italian patients.

## 2. Results

All the single-nucleotide polymorphisms analyzed in this study (Table 2) were successfully genotyped in all subjects (N = 298) by targeted next-generation sequencing. The obtained genotype for each cytochrome was converted to a predicted phenotype and the patient was classified, accordingly, as a normal metabolizer (NM), poor metabolizer (PM), intermediate metabolizer (IM), or rapid/ultra-rapid metabolizer (UM).

**Figure 1:**
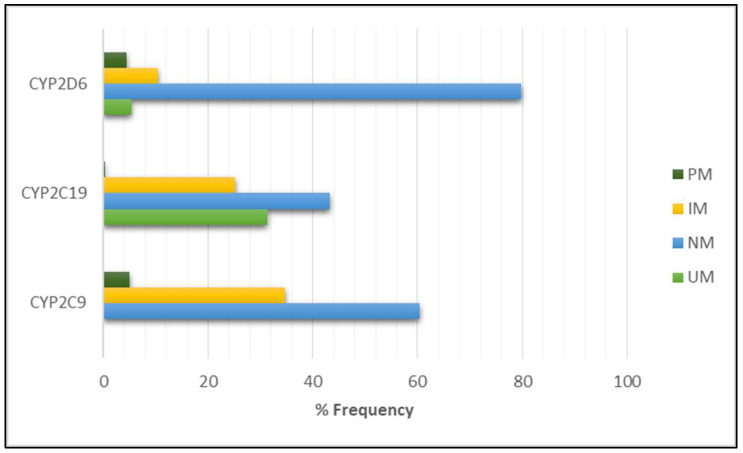
Frequencies of the normal (NM), intermediate (IM), poor (PM), and ultra-rapid (UM) metabolic phenotypes for CYP2D6, CYP2C19, and CYP2C9.

The genotype results and the associated predicted phenotype for each patient are reported in Supplementary Table S1. The phenotype frequencies for each cytochrome are shown in Figure 1. Concerning CYP2C9, 180 (60.4%) subjects were classified as NM, 103 (34.6%) subjects were classified as IM, and 15 (5%) subjects were classified as PM. Regarding CYP2C19, 129 (43.3%) subjects were classified as NM, 93 (31.2%) subjects were classified as UM, 75 (25.2%) subjects were classified as IM, and 1 (0.3%) subject was classified as PM. Regarding CYP2D6, 238 subjects (79.96%) were classified as NM, 31 (10.4%) subjects were classified as IM, 16 (5.3%) subjects were classified as UM, and 13 (4.36%) subjects were classified as PM.

Thus, in the analyzed population, alterations in the CYP2C19 phenotype are the most frequent, with a total of 56.7% of subjects with an IM, PM, or UM phenotype, followed by alterations in the CYP2C9 phenotype (39.6% subjects with an IM or PM phenotype) and alterations in the CYP2D6 phenotype (20.1% subjects with an IM, PM, or UM phenotype).

**Figure 2:**
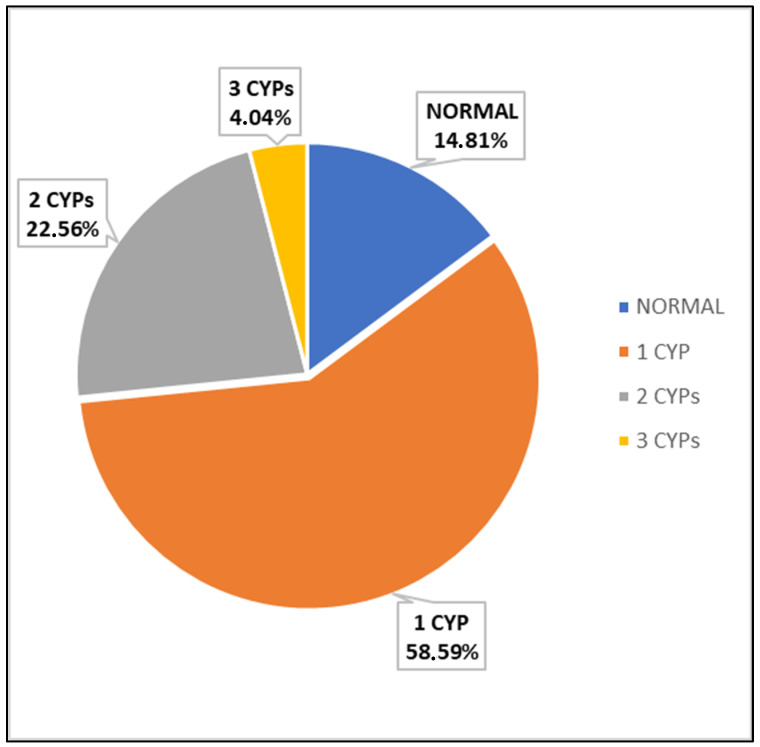
Frequencies of subjects carrying 1, 2, or 3 dysfunctional CYP450s.

We then analyzed the frequency of subjects carrying more than one dysfunctional cytochrome (CYP2C9, CYP2C19, or CYP2D6), defining a CYP450 dysfunction as the presence of a non-normal cytochrome phenotype (any of IM, PM, or UM). We found that just 44 out of the 298 (14.8%) subjects have three fully functional CYP450 isozymes, whereas 174 (58.6%) subjects have at least one dysfunctional CYP450, 67 (22.5%) subjects have two dysfunctional CYP450s, and 12 (4%) subjects have three dysfunctional CYP450s (Figure 2). Table 3 reports the ranked frequencies of combined CYP450 phenotypes. The most frequent combined phenotypic dysfunctions are represented by the alteration of both CYP2C9 and CYP2C19 activity (10.05%), the alteration of both CYP2C19 and CYP2D6 activity (7.05%), and the alteration of both CYP2C9 and CYP2D6 activity (5.36%).

## 3. Discussion

In the last decade, PGX testing has developed patchily due to cost-effectiveness considerations and to healthcare reimbursement policies in different countries. Nowadays, testing costs are decreasing, and PGXs could be applied more systematically.

In this study, we show that only around 14% of the analyzed subjects have fully functional CYP2C9, 2C19, and 2D6 (based on the analyzed gene variants). Further, more than one-quarter of the patients have at least two dysfunctional (UM, PM, or IM) CYP450s (Figure 2). In our opinion, these merely descriptive data are deeply relevant to fully understanding the unseen potential of more systematic and comprehensive PGX testing. To support this suggestion, we report in Table 4 the top 20 prescribed drugs in the USA in 2020 [22]; most of them are often co-prescribed, as they are used to treat frequent comorbidities such as cardiovascular diseases, hypertension, metabolic disorders, and gastric-acid-related disorders [23,24,25]. Looking at the drug–CYP450 interactions reported in Table 4, and keeping in mind the frequency of combined CYP450 dysfunction, it seems apparent how improper prescription can easily occur, especially in the cases of co-prescription or polytherapy (five or more drugs) prescription.

Polytherapy regimens are a well-established factor associated with increased rates of ADRs, hospitalization, nonadherence, and death [26,27,28,29,30]. The USA Centers for Disease Control and Prevention reported that in the period 2015–2018, 24% of persons had been taking three or more drugs in the past month, and 12.8% of persons had been taking five or more drugs in the past month [31]. Sutherland et al., in an interesting analysis of medication co-prescription patterns in more than 10.000 patients in the USA, found that more than 20% of over-65s were taking drugs that are both substrates and inhibitors of CYP450s [23]. The significant demographic shift towards higher ages (2.1 billion over-60s and 426 million over-80s by 2050, according to WHO) [32] means an increasing number of multimorbid patients and an increasing pharmaceutical expense [33].

The societal challenge of healthy aging requires a drastic improvement in the safety and efficacy of pharmacological treatments, as well as an improvement in compliance and adherence to the prescribed therapies. Supporting appropriate therapy selection by DDI/PGX evaluation could greatly contribute to such demand, and the strategy is supposed to be cost-effective if applied to a well-defined patient population such as the elderly, or generally in polytreated patients. In this regard, let us mention the categories of oncologic and psychiatric patients.

Although oncology has always been a main field for precision medicine development and implementation, PGX testing in the field is usually targeted to predict response to the main antitumor medication(s). But cancer patients are frequently co-prescribed non-oncologic medications (often analgesics and antiemetics); further, cancer prevalence is higher in aged people already receiving treatment for comorbidities [34,35,36]. The reciprocal interaction effect of oncologic and non-oncological medications is rarely considered, while it brings the potential to interfere with anticancer therapy. It is estimated that about 30% of cancer patients have a significant risk of severe DDIs [37], most of them involving non-oncological medications such as coumarin, antiepileptics, and opioids [38].

In psychiatry, polypharmacy represents a standard treatment and includes several drug classes such as antidepressants, antipsychotics, mood stabilizers, anxiolytics, hypnotics, antihistamines, and anticholinergics [39,40]. About one-third of these patients experience severe side effects [39]. The optimization of drug cocktails in these patients according to PGXs and DDIs has been proven to be effective in ameliorating the treatment outcomes [41,42,43].

Recently, the Dutch Pharmacogenetics Working Group (DPWG) reported a nation-wide evaluation of the cost-effectiveness of systematic adoption of a prescription decision algorithm based on a single gene PGX test. The authors estimated that properly prescribed single gene testing may prevent a considerable number of deaths per year due to drug therapy failure [44]. Thus, it can be reasonably supposed that the number of avoidable treatment failures (in terms of safety/efficacy) may be significantly decreased by the appropriate prescription of comprehensive (multigene) PGX testing.

Given the limits of the present study, we believe that the observed extent of combined dysfunction in different CYP450s constitutes a non-negligible reason to design shared guidelines supporting the appropriate and systematic prescription of PGX testing, and that healthcare policy makers should start to pay more attention to the unseen potential of DDI analysis/PGX testing in polytreated patients [45,46].

The abovementioned limits of our study include the restricted number of samples and the recruiting environment of the participants, e.g., the Unit of Psychiatry of the Sant’Andrea University Hospital of Rome, since it may have introduced a bias in the observed distribution of CYP450 phenotypes. This could arise from an increased willingness to participate in the study by people who experienced poor therapy efficacy and compliance, a common situation in psychiatric patients [47,48]. On the other hand, this could also be regarded as a point of strength, considering our intent to support the importance of pre-emptive PGX testing in fragile, polytreated patients.

We also want to mention, as a limit shared by our study and by the overall PGX community, that there is a certain degree of uncertainness in the genotype–phenotype conversion that may account for some discrepancy in the CYP450 phenotype definitions reported by different researchers. The reasons for such uncertainness have been extensively discussed elsewhere [49,50,51,52], but they include technological issues, the limited portion of genomic variation screened by PGX assays, and some discordance/lack of full evidence regarding the actual quantitative functional effect of gene polymorphisms on the actual enzyme activity/expression.

## 4. Materials and Methods

### 4.1. Population

PGX testing was performed on consecutive patients (N = 298, 174 females and 124 males, mean age 47.7 ± 28.9 years) referred to the Unit of Psychiatry of the Sant’Andrea University Hospital of Rome, Italy, in the period 2018–2023. The study was approved by the local ethical committee (protocol N. 6279/2021) and performed according to the Principles of Human Rights adopted by the World Medical Association (WMA) at the 18th WMA General Assembly, Helsinki, Finland, in June 1964, and subsequently amended by the 64th WMA General Assembly, Fortaleza, Brazil, in October 2013. All participants gave their informed written consent.

### 4.2. PGX Testing

PGX testing was performed on a genomic DNA sample obtained from peripheral, EDTA anticoagulated blood (3 mL). DNA was purified from 200 μL of whole blood using the Qiasymphony automated nucleic acid extraction system (Qiagen, Hilden, Germany) with a DSP DNA mini kit (Qiagen, Hilden, Germany), according to the manufacturer’s instructions.

A custom targeted-sequencing NGS panel was purchased from ThermoFischer Scientific (Waltham, MA, USA). The NGS assay panel consisted of a pool of primer pairs specifically designed by the vendor to target DNA regions of interest, including the genomic coordinates of the polymorphisms listed in Table 2. The pool of primers was used to amplify genomic DNA and prepare DNA libraries using the Ion AmpliSeq™ Library Kit 2.0 (ThermoFisher Scientific, Waltham, MA, USA), according to the manufacturer’s instructions. Clonal amplification, chip loading, and sequencing were performed on the IonChef/IonS5 GeneStudio system (ThermoFisher Scientific, Waltham, MA, USA), using the Ion 510™ & Ion 520™ & Ion 530™ Kit—Chef according to the manufacturer’s instructions (ThermoFisher Scientific, Waltham, MA, USA). Sequencing data were processed using the IonReporter software (ThermoFisher Scientific, Waltham, MA, USA), which embedded a bioinformatics pipeline allowing automatic variant calling and copy number variation evaluation.

### 4.3. Genotype-Based Prediction of CYP450 Phenotype

Dysfunctional alleles were defined as alleles giving a known alteration of protein function, according to the Clinical Pharmacogenetics Implementation Consortium (CPIC) [4,18,19,20,21]. The CPIC’s guidelines assign to each polymorphic allele an activity score (AS) ranging from 0, indicating a no-function allele, to 1, indicating a normal functioning allele. Genotype–phenotype translation is performed as follows: subjects with AS = 0 (two null alleles) are poor metabolizers (PMs); subjects with AS = 0.25–1 (two reduced-function alleles or one null allele plus a normal allele) are intermediate metabolizers (IMs); subjects with AS = 1.25–2 are normal metabolizers (NMs); and subjects with AS > 2.25 (copy number variation giving an allele duplication) are ultra-rapid metabolizers (UMs).

### 4.4. Statistics

Descriptive statistics were calculated using the SPSS software version 25 (IBM Statistics). To analyze combined cytochrome phenotype frequencies, subjects characterized as PM, IM, and UM were grouped together as “dysfunctional CYP450(s)” carriers.

## 5. Conclusions

The low frequency of subjects with a fully functional set of cytochrome P450 enzymes claims a paradigm shift in the approach to PGX test prescription, also considering the increasing sustainability and availability of next-generation sequencing, allowing the assessment of many pharmacogenes in the same test [53]. Since the additional healthcare expenditure associated with increased rates of ADRs, hospitalization, and death in polytreated patients is well documented, it is expected that the cost of preemptive and comprehensive PGX testing is fully affordable when considering the benefits, at least in such patient groups.

## Figures and Tables

**Table 1:** CYP450 pharmacogenomic biomarkers in drug labels, by the FDA. The numbers of labelled drugs (N) and their therapeutic areas are shown.

CYP2C9 (N = 17)	CYP2C19 (N = 25)	CYP2D6 (N = 72)	CYP2B6 (N = 3)	CYP3A5 (N = 1)	CYP1A2 (N = 1)
Anesthesiology		Anesthesiology			
Cardiology	Cardiology	Cardiology	Cardiology	Cardiology	
		Dental			
	Dermatology				
Endocrinology					
Gastroenterology	Gastroenterology	Gastroenterology			
Gynecology	Gynecology	Gynecology	Gynecology		
Hematology					
		Inborn Errors of Metabolism			
	Infectious Diseases	Infectious Diseases	Infectious Diseases		
Neurology	Neurology	Neurology			
Oncology	Oncology	Oncology			Oncology
	Psychiatry	Psychiatry			
	Pulmonary	Pulmonary			
Rheumatology	Rheumatology	Rheumatology			
		Urology			

**Table 2:** List of tested gene variants and allele functional status according to the CPIC allele functionality reference tables [18,19,20,21].

Analyzed Gene Polymorphisms	Functional Status
CYP2C9	
*2	Decreased function
*3	No function
CYP2C19	
*2, *3, *4, *5, *7	No function
*10	Decreased function
*17	Increased function
CYP2D6	
*2, *2A	Normal function
*3, *4, *5, *6, *7, *20, *38	No function
*9, *10, *41	Decreased function
*17, *29*	Decreased function

**Table 3:** Ranked frequencies of combined CYP450 phenotypes (N = 298).

CYP2C9 Phenotype	CYP2C19 Phenotype	CYP2D6 Phenotype	N	%
NM	UM	NM	55	18.46
IM	NM	NM	50	16.78
NM	IM	NM	49	16.44
NM	NM	NM	44	14.80
IM	UM	NM	17	5.70
IM	IM	NM	11	3.69
PM	NM	NM	9	3.02
IM	UM	IM	7	2.35
NM	NM	IM	7	2.35
IM	NM	IM	5	1.68
IM	NM	UM	5	1.68
NM	IM	IM	4	1.34
NM	IM	PM	4	1.34
NM	UM	UM	4	1.34
IM	NM	PM	3	1.01
IM	IM	IM	3	1.01
NM	IM	UM	3	1.01
NM	UM	IM	3	1.01
NM	UM	PM	3	1.01
NM	NM	PM	2	0.67
PM	NM	UM	2	0.67
PM	UM	NM	2	0.67
IM	IM	UM	1	0.34
IM	UM	PM	1	0.34
NM	NM	UM	1	0.34
NM	PM	NM	1	0.34
PM	NM	IM	1	0.34
PM	UM	IM	1	0.34

**Table 4:** List of the 20 most prescribed medications. Drug interactions with CYP2C19, CYP2C9, and CYP2D6 and therapeutic categories/areas are shown.

Medication	CYP2C19	CYP2C9	CYP2D6	Therapeutic Category	Therapeutic Area
Atorvastatin	Inh	Inh	Inh	Antihyperlipidemic agents	Metabolic disease
Levothyroxine				Thyroid drugs	Hypothyroidism
Metformin				Antidiabetic agents	Metabolic disease
Lisinopril				Angiotensin-converting enzyme inhibitors	Cardiovascular disease
Amlodipine		Inh	Inh	Calcium channel blocking agents	Cardiovascular disease
Metoprolol	S		Inh S	Beta-adrenergic blocking agents	Hypertension
Albuterol				Bronchodilators	Respiratory diseases
Omeprazole	Ind Inh S	Inh S	Inh	Proton pump inhibitors	Gastric-acid-related disorders
Losartan	Inh	Inh S		Angiotensin II inhibitors	Hypertension
Gabapentin				Anticonvulsants	Neuroleptic agents
Hydrochlorothiazide				Diuretics	Cardiovascular disease
Sertraline	Inh S	Inh S	Inh S	Antidepressants	Psychotherapeutic agents
Simvastatin	Inh S	Inh	Inh S	Antihyperlipidemic agents	Metabolic agents
Montelukast		S		Bronchodilators	Respiratory diseases
Escitalopram	Inh S		Inh S	Antidepressants	Psychotherapeutic agents
Acetaminophen		S	Ind S	Analgesic	Analgesic
Rosuvastatin		Inh S		Antihyperlipidemic agents	Metabolic agents
Bupropion	S	S	Inh S	Antidepressants	Psychotherapeutic agents
Furosemide				Diuretics	Cardiovascular disease
Pantoprazole	Inh S	Inh		Proton pump inhibitors	Gastric-acid-related disorders
Aspirin	Ind	S	Inh	Analgesic and antiplatelet agents	Analgesic/Cardiovascular disease

## Data Availability

Data is contained within the article or Supplementary Material.

## Supplementary Materials

The following supporting information can be downloaded at: https://www.mdpi.com/article/10.3390/ijms241612696/s1.
